# The *Streptococcus sanguinis* Competence Regulon Is Not Required for Infective Endocarditis Virulence in a Rabbit Model

**DOI:** 10.1371/journal.pone.0026403

**Published:** 2011-10-19

**Authors:** Jill E. Callahan, Cindy L. Munro, Todd Kitten

**Affiliations:** 1 VCU Philips Institute of Oral and Craniofacial Molecular Biology, Virginia Commonwealth University, Richmond, Virginia, United States of America; 2 Department of Microbiology and Immunology, Virginia Commonwealth University, Richmond, Virginia, United States of America; 3 Department of Adult Health Nursing, Virginia Commonwealth University, Richmond, Virginia, United States of America; 4 Center for the Study of Biological Complexity of Virginia Commonwealth University, Richmond, Virginia, United States of America; National Institutes of Health, United States of America

## Abstract

*Streptococcus sanguinis* is an important component of dental plaque and a leading cause of infective endocarditis. Genetic competence in *S. sanguinis* requires a quorum sensing system encoded by the early *comCDE* genes, as well as late genes controlled by the alternative sigma factor, ComX. Previous studies of *Streptococcus pneumoniae* and *Streptococcus mutans* have identified functions for the >100-gene *com* regulon in addition to DNA uptake, including virulence. We investigated this possibility in *S. sanguinis*. Strains deleted for the *comCDE* or *comX* master regulatory genes were created. Using a rabbit endocarditis model in conjunction with a variety of virulence assays, we determined that both mutants possessed infectivity equivalent to that of a virulent control strain, and that measures of disease were similar in rabbits infected with each strain. These results suggest that the *com* regulon is not required for *S. sanguinis* infective endocarditis virulence in this model. We propose that the different roles of the *S. sanguinis*, *S. pneumoniae*, and *S. mutans com* regulons in virulence can be understood in relation to the pathogenic mechanisms employed by each species.

## Introduction

Apart from caries formation by Mutans group species, oral streptococci generally play a benign or beneficial role within the oral cavity. Because of their habitat and the rich vasculature of the gingival tissue, however, the oral streptococci may be seeded into the bloodstream through oral surgery or everyday activities including brushing and chewing [1], [2]. Blood-borne bacteria may colonize endocardium or cardiac valves that have been damaged by congenital conditions or degenerative processes, resulting in infective endocarditis (IE) [3], [4]. IE is fatal if not treated with antibiotics or surgery. Oral streptococci are a common cause of IE, with *Streptococcus sanguinis* frequently reported as the most commonly isolated oral species [5]. *S. sanguinis* virulence for IE has been examined in a number of studies [6]–[11] but very few genes or functions required for virulence have been identified. This may be due to functional redundancy of some virulence factors [11]. If so, virulence reduction could require simultaneous elimination or downregulation of multiple genes.

*S. sanguinis* exhibits genetic competence [12], [13], the ability to acquire and incorporate exogenous DNA into its genome. This capability also occurs in certain other streptococcal species, and is best characterized in *Streptococcus pneumoniae*, in which it was first discovered. In this species, a number of “early” *com* genes are essential for induction and regulation of this cascade [14]. The *comC* gene encodes a precursor peptide that is cleaved and exported by the ComA and ComB proteins. The mature ComC peptide, termed “competence-stimulating peptide” (CSP), is sensed by the ComD sensor kinase, which is thought to respond by phosphorylating the ComE response regulator. The latter two proteins are encoded along with ComC in the *comCDE* operon. ComE then activates expression of the *comAB* and *comCDE* operons. These genes therefore form a quorum sensing system, which results in amplification of the component gene products when the concentration of extracellular CSP reaches a critical density. ComE also activates expression of the *comX* gene, which is present in two copies. ComX serves as an alternative sigma factor, directing the expression of “late” genes required for uptake and incorporation of DNA. ComW serves to stabilize ComX and also activates it by an unknown mechanism.

Despite the apparent absence in *S. sanguinis* of the *comA*, *comB*, and *comW* genes [15], physiological studies and global expression analyses have demonstrated that *S. sanguinis* competence and *com* gene regulation are similar in most respects to that of *S. pneumoniae* [13]. Microarray and RT-PCR studies performed with a *comC* mutant suggested upregulation of 124 genes in *S. sanguinis* in response to CSP [13]. A microarray analysis performed with *S. pneumoniae* [16] identified a similar, though non-identical set of CSP-upregulated genes [13].

Roles for the streptococcal *com* regulon apart from genetic competence have been postulated based on the finding in *S. pneumoniae* that all but 23 of the 124 upregulated genes were individually dispensable for transformation [16]. Previous studies of systemic disease in *S. pneumoniae* [17]–[19] and caries formation by *S. mutans* [20] have indicated that *com* regulon genes contribute to virulence in these species by mechanisms that are likely unrelated to DNA uptake. Here we report the creation of mutants with deletions in key regulators of the *S. sanguinis com* regulon, and their examination in a rabbit model of IE. Our results suggest that in contrast to a number of *S. pneumoniae* and *S. mutans* strains that have been examined, *S. sanguinis* SK36 does not require induction of the *com* regulon for virulence. This difference may be explained by characteristics of *S. pneumoniae* and *S. mutans* virulence that are not shared by *S. sanguinis*.

## Materials and Methods

### Ethics Statement

All animal procedures were approved by the Institutional Animal Care and Use Committee of Virginia Commonwealth University (permit AM10030) and were compliant with all institutional policies and federal guidelines, including the USDA Animal Welfare Act and the Public Health Service Policy on the Humane Care and Use of Laboratory Animals.

### Bacterial strains and growth conditions

*S. sanguinis* SK36, isolated from human dental plaque [15], [21], was used in these studies. Strains derived from SK36 and plasmids used in this study are listed in Table 1. SK36 and derivates were grown at 37°C in Anoxomat jars (Spiral Biotech) under microaerobic conditions (7% H_2_, 7% CO_2_, 80% N_2_, and 6% O_2_) in brain-heart infusion (BHI; Bacto, Sparks, MD) broth or on BHI plates with 1.5% agar, except as noted below. For transformations, *S. sanguinis* strains were grown in Todd Hewitt broth (Bacto, Sparks, MD) containing 2.5% horse serum (Invitrogen) (THHS) [8], [22] and incubated with transforming DNA. For streptococcal selection, chloramphenicol (Cm), erythromycin (Em), spectinomycin (Sc) and kanamycin (Kn) were employed at 5 µg/ml, 10 µg/ml, 200 µg/ml, and 500 µg/ml, respectively.

**Table 1:** Strains and plasmids used in this study.

Strain or plasmid	Description	Reference
*S. sanguinis* strains		
SK36	Human plaque isolate	[15], [21]
JFP36	Em^r^; SSA_0169::pSerm; derived from SK36	[22]
JFP45	Kn^r^; Δ*comX*::*aphA-3*; derived from SK36	This study
JFP49	Sc^r^; Δ*comCDE*::*aad9*; derived from SK36	This study
Plasmids		
pJFP16	Kn^r^ Cm^r^; pVA2606 containing 3.3-kb *nrdD1::magellan2*	[22]

### Creation of mutants

Deletion of the *comCDE* operon was performed using an overlap extension PCR technique [23], in which the *comCDE* operon was replaced with the *aad9* Sc^r^ cassette of pR412 [24]. Briefly, three separate PCR reactions were performed. The comCDEup1 and comCDEdn2 primers were used for amplification of the 1-kb region upstream of *comCDE*, the comCDEup3 and comCDEdn4 primers for the *aad9* cassette, and the comCDEup5 and comCDEdn6 primers for the 0.8-kb region downstream from the operon. (See Table 2.) In these reactions, primers were designed to create 25-bp regions of complementarity between the 3′ end of the upstream region and 5′ end of the *aad9* cassette, and between the 3′ end of the *aad9* cassette and the 5′ end of the downstream region. A fourth PCR using primers comCDEup1 and comCDEdn6 fused the three PCR products. All reactions were performed using a high-fidelity polymerase Supermix (Invitrogen). The 2.5-kb PCR fusion product was column-purified (Qiagen, Inc. Valencia, CA) and used to transform SK36, with Sc selection. DNA sequencing confirmed that the mutant, JFP49, contained the expected *comCDE* deletion and Sc^r^ cassette insertion, and had flanking sequences identical to the SK36 published sequence (accession number NC_009009) as expected.

**Table 2:** Primers used in this study.

Primer	Nucleotide sequence (5′ to 3′)
comCDEup1	CCTCCTTGAATTACAATTGATG
comCDEdn2	GGATCCACTAGTTCTAGAGCGGAACTATCTCCTATCTTTTTATCTT
comCDEup3	AAGATAAAAAGATAGGAGATAGTTCCGCTCTAGAACTAGTGGA
comCDEdn4	TTCCATTATATCAGGTTTCAATTTTTTTATAATTTTTTTAATCTG
comCDEup5	CAGATTAAAAAAATTATAAAAAAATTGAAACCTGATATAATGGA
comCDEdn6	AGCTGGCTATTTCAGTCAAAGTC
comXkanF	GCTTTTTAGAAGAAAGAGCCCACCTTGCTTTCTATTTACAGTAGAATTAAGTCAAGTAAATTTTAAGGAGGAACTAAGGGCCCGTTTGAT
comXkanR	TTTTTATTTAATAAAAAAACTTGATGCTCTAACCTGTTCTAGTAAAACAGAAATAAAGTCATCAAGTTTGTTCACCGAATTCTAGGTACT

The *comX* gene was replaced with the *aphA-3* gene encoding Kn^r^ [25] using a novel, long-primer approach. Briefly, the *aphA-3* gene was amplified using 90-nt primers constructed to possess 15-nt 3′ ends complementary to the beginning and end of the *aphA-3* gene and 75-nt 5′ ends complementary to the regions immediately upstream and downstream of *comX* (Table 2). The PCR product was purified as above and used to transform SK36, with selection for Kn^r^. DNA sequencing confirmed that the mutant, JFP45, contained the desired cassette insertion and the sequences expected for SK36 within the flanking regions.

### Transformation Assays

Transformation was used in mutant generation and as a measure of competence using a modification of a previously described transformation assay [13]. Briefly, overnight cultures of each strain exposed to ambient atmosphere were diluted 1∶200 into prewarmed THHS and incubated at 37°C until reaching an OD_660_ of approximately 0.07. Aliquots of 330 µl were added to prewarmed 0.7-ml microcentrifuge tubes containing ±70 ng synthetic *S. sanguinis* CSP [12] and 10 ng pJFP16 [22], which is a suicide plasmid encoding Cm resistance, and incubated at 37°C for 60 to 90 min. Dilutions were then drop-plated [26] onto BHI agar plates and enumerated. Transformation frequency was measured following 48 h incubation as total CFU/ml on BHI plates containing Cm divided by total CFU/ml from plates with no antibiotics.

### Virulence assays

Virulence assays were performed using a rabbit aortic valve endocarditis model employing specific-pathogen-free, male New Zealand White rabbits (RSI Biotechnology, Mocksville, N.C.) weighing 3 to 4 kg, as described previously [8]–[11], [22]. In brief, a small incision was made in the neck of each anesthetized animal to expose the right carotid artery. To induce formation of a sterile vegetation [27], the artery was tied off at the cephalic end, and a sterile 19-gauge catheter (BD, Sandy UT) was threaded through a nick in the artery until resistance was met, indicating contact with the aortic valve or left ventricle. The catheter was then trimmed, ligated, and sutured in place, and the incision closed with surgical clips.

To prepare inocula, bacterial strains were grown overnight in BHI, and diluted 10-fold into BHI for an additional 3 h of growth at 37°C. Cells were then harvested, washed in PBS, and diluted to the appropriate cell density, followed by inoculation of 0.5 ml into a marginal ear vein. Inoculations were performed two days post-catheterization. Dilutions of the inocula were drop-plated onto BHI agar, with selective antibiotic when required, and enumerated. After specified infection periods, rabbits were euthanized by intravenous Euthasol (Virbac AH, Fort Worth, TX) injection. Hearts were removed and correct catheter placement was verified. Vegetations, which were readily identified as rough nodules or masses, were collected from aortic valves and endocardium and homogenized in PBS. Dilutions were drop-plated for bacterial enumeration.

For competitive index (CI) experiments, JFP45 or JFP49 was co-inoculated with an Em^r^ derivative of SK36, JFP36 [22], and the strains were co-cultured for 3 h prior to washing and suspension in PBS, as described above. The CI value for each rabbit was calculated as the ratio of mutant/JFP36 in the vegetation homogenate divided by the mutant/JFP36 ratio in the inoculum.

For experiments employing inoculation of individual strains, inocula were prepared as described above, except that strains were cultured individually, randomized for individual inoculation into four catheterized rabbits each, and sonicated prior to inoculation at 50% power for 3 minutes using a titanium cup adapter (BioLogics Inc.) Following sonication, cells were adjusted to an OD_660_ value determined previously to correspond to 10^8^ CFU/ml. During the course of infection, rabbits were weighed daily. Five days post-inoculation, ∼10 ml of blood was drawn from an ear artery of surviving rabbits using a blood collection set (BD, Franklin Lakes, N.J.; cat. no. 368653) and blood culture tubes containing 1.7 ml of 0.35% sodium polyanethol sulfonate solution (BD; cat. no. 364960). Rabbits were immediately sacrificed, necropsy was performed, and valve vegetations were weighed prior to homogenization and sonication. Density of infection was determined as CFU/g of vegetation. Blood densities were determined by mixing 10 µl to 1 ml of blood with 20 ml 1% low-melting agarose in BHI, which was then poured into Petri dishes, allowed to harden, and incubated for two days at 37°C prior to counting of colonies on triplicate plates. Blood density values were corrected to account for the added sodium polyanethol sulfonate. The entire experiment was then repeated on a separate occasion, and the results combined.

Efforts were made to ameliorate suffering of animals at all stages of the experiment. Prior to surgery, animals were sedated with acepromazine, anesthetized with a cocktail of ketamine, xylazine, glycopyrrolate, and buprenorphine, and injected with bupivacaine at the incision site. Acepromazine was also provided prior to blood collection and euthanasia. Buprenorphine was provided every 12 h as analgesia from the time of surgery until euthanasia.

### Statistical Tests

All statistical tests were performed using InStat (GraphPad Software Inc.), with α = 0.05. For virulence assays, CI values were compared to 1.0 using the Wilcoxon rank sum test. Strain recoveries, infection densities, vegetation masses, and weight loss for each strain group were compared using the Kruskal-Wallis test. Survival rates were compared by Kaplan-Meier analysis. Median and interquartile values were calculated using JMP software (SAS Institute, Inc.).

## Results

### Creation and verification of *com* mutants in *S. sanguinis*

To examine the role of the *S. sanguinis com* regulon in virulence, we first sought to create mutations in key regulatory genes. We used an overlap extension PCR technique [23] to fuse a Sc^r^ cassette to ∼1 kb of DNA on either side of the *comCDE* operon. Introduction of this construct into SK36 resulted in the replacement of the *comCDE* genes with the Sc^r^ cassette, creating mutant JFP49. (See Table 1 for strains and Table 2 for primers.) Mutation of the *comX* gene by the same strategy was problematic because the gene is closely flanked by tRNA genes on one side and a 16S rRNA gene on the other [15]. To avoid transforming with a construct containing these repetitive sequences, we attempted the novel approach of amplifying a Kn^r^ gene [25] with PCR primers designed with 75-nt 5′ ends complementary to *comX*-flanking sequences, and then introducing the purified product into SK36 by transformation. Although the transformation efficiency was low, we succeeded in isolating a mutant, JFP45, in which the *comX* gene was replaced by the Kn^r^ cassette.

We then tested both mutants in transformation assays using a Cm resistance-encoding suicide plasmid, pJFP16 [22]. We expected that the *comCDE* mutant would not undergo transformation in either the presence or absence of added synthetic CSP, as loss of the CSP sensor and its cognate response regulator would not be overcome by the addition of CSP [16]. As expected, mutation of *comCDE* drastically reduced or eliminated transformation, lowering the frequency from 12% in SK36 to below the level of detection (1.3×10^−5^%) in JFP49 in both the presence and absence of CSP. Similarly, we expected a *comX* mutant to exhibit little or no competence in the presence or absence of CSP, as the sigma factor it encodes is required for upregulation of DNA uptake genes in other species [16]. Comparable results were obtained; deletion of *comX* lowered the transformation frequency from 12% in SK36 to below the limit of detection (7.7×10^−5^%) in JFP45, in the presence or absence of CSP. The combined results suggest that these four genes function in *S. sanguinis* competence regulation indistinguishably from their counterparts in *S. pneumoniae* [16], *S. gordonii* [28], and other Mitis group species [29].

### Examination of *com* regulatory gene mutants for IE virulence in competition assays

**Figure 1:**
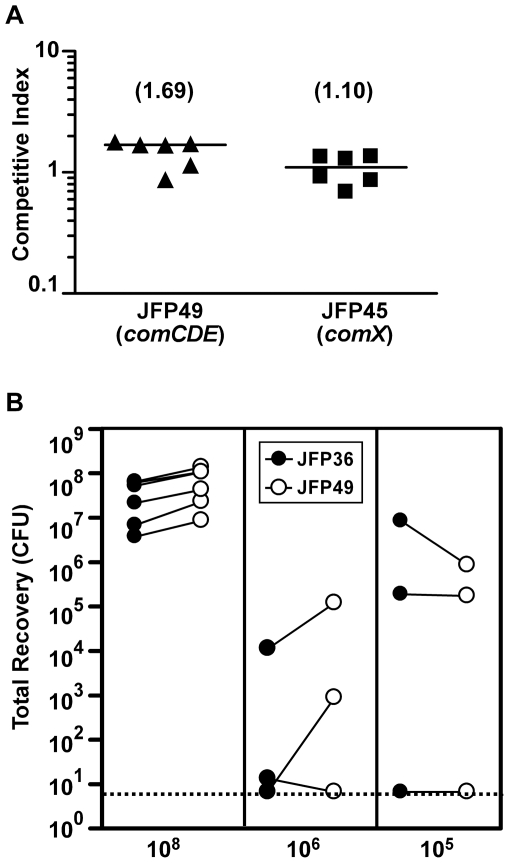
Examination of *com* mutants by competitive index analysis in a rabbit model of endocarditis. Each symbol represents data from a single rabbit. (A) CI values from rabbits inoculated with 10^8^ CFU. Median CI values from the combined results of two experiments are indicated in parentheses and by horizontal bars. The CI value for each rabbit was calculated as the ratio of mutant/JFP36 in the vegetation homogenate divided by the mutant/JFP36 ratio in the inoculum. Neither median value was significantly different from 1.0. (B) Recovery of competing strains from infected vegetations at multiple inoculum levels. Each pair of connected circles indicates recovery of JFP36 (filled circles) and JFP49 (open circles) from the same rabbit. Data for the 10^8^ inoculum are from experiments depicted in panel A. Dashed line, limit of detection.

Once we had confirmed that our *com* regulatory mutants had the expected phenotypes, we wanted to determine if the *com* regulon contributes to IE virulence in *S. sanguinis*. We began by testing the *comCDE* and *comX* mutants by competitive index (CI) assays in our standard rabbit endocarditis model [8]–[11], [22]. Rabbits catheterized to induce sterile vegetations were co-inoculated with ∼10^8^ CFU of one of the mutants and JFP36—an Em^r^ derivative of SK36 that we have shown to be indistinguishable in multiple traits, including growth rate, genetic competence, and virulence for IE [22]. CI values for each rabbit are presented Fig. 1A. Median CI values for both mutants were slightly greater than 1.0, although neither difference was statistically significant (*P* = 0.063 for the *comCDE* mutant and 0.56 for the *comX* mutant). The results suggested that neither *comCDE* nor *comX* was required for IE virulence in our standard assay.

Previous studies of IE in the *S. gordonii* rat model have demonstrated increased sensitivity in competition assays when inoculum levels were reduced [30], [31]. To determine whether this might increase our ability to detect subtle changes in competitiveness, we tested lower co-inoculum levels (10^5^ and 10^6^) of JFP36 and the *comCDE* mutant in three rabbits each. In contrast to our previous results, these experiments revealed marked variability in individual CI values at both inoculum levels. Indeed, CI values could not be calculated for one rabbit in each group, due to a failure to recover JFP49 from a rabbit with the 10^5^ inoculum and JFP36 from a rabbit with the 10^6^ inoculum. This is illustrated in Fig. 1B, which shows the number of JFP36 and JFP49 CFU recovered in each experiment. As seen, the use of lesser inocula generally resulted in lower and more variable recoveries of both strains, suggesting a bottleneck effect, whereby too few cells establish infection to ensure accurate representation of each strain [8], [11], [32]. In contrast, use of a 10^8^ inoculum resulted in highly reproducible recovery of both strains. We therefore concluded that lower inoculum levels could not be used as a method for increasing the sensitivity of our CI assays.

### Examination of *com* regulon mutants for IE virulence and pathogenesis by individual inoculation

Our finding that neither *com* regulatory mutant showed attenuation of IE virulence prompted us to consider other modifications to our virulence assay. First, we considered the possibility that strains might be affected by co-inoculation, with one strain producing, for example, a trans-acting factor that had an effect on the other. This possibility could be eliminated by inoculating mutant and control strains into separate animals. In addition, this would allow for comparison of the pathology caused by each strain. We also considered the possibility that a longer infection period would reveal a contribution of the *com* regulon to virulence that was not apparent from the 20-h infection employed in our previous assays. This, too, would represent a relevant condition, since streptococcal IE typically progresses for days or weeks before detection and treatment [3], [4]. Finally, we also considered the possibility that virulence differences might be obscured by variation in colony counts caused by chain length variation, as mutation of *comX* resulted in longer chains relative to JFP36 (data not shown). We simultaneously tested all three possibilities by sonicating strains prior to inoculation and after recovery from the animal to eliminate potential chain-length differences, inoculating mutant strains and JFP36 into separate rabbits, and extending the infection time to 5 days.

A total of eight rabbits each were inoculated with 10^8^ CFU of the *comCDE* or *comX* mutants, or JFP36. One rabbit inoculated with JFP36 yielded only a gram-negative rod, in low numbers (∼300 CFU compared to >10^7^ CFU of streptococci recovered from each of the other rabbits). It is not clear whether these bacteria were present in the heart valve or were introduced during sample collection; nevertheless, this animal was removed from the analysis. Two animals each that were inoculated with the *comCDE* or *comX* mutants died prematurely on day 4. Results from the remaining animals are presented in Table 3. The total number of CFU recovered from vegetations for each of the three strains did not differ significantly. We next compared recovery in terms of bacterial density within vegetations. No significant differences were observed when the recovery levels were normalized by vegetation mass. We also compared bacterial levels in the blood just prior to sacrifice. There was also no significant difference in bacteremia levels among the three strains. Although the data are not included in Table 3, we were also able to recover vegetations within 2 h of death for two of the animals that died on day 4. The bacterial recoveries (1.4×10^9^ CFU and 3.8×10^9^ CFU/g for the *comCDE*-inoculated animal and 2.3×10^9^ CFU and 4.6×10^9^ CFU/g for the animal inoculated with the *comX* mutant) were similar to those from animals that survived until day 5. Inclusion of these data along with those in Table 3 in the statistical analysis did not change the result—there were still no significant differences in total CFU or CFU/g among the three groups.

**Table 3:** Recovery of *S. sanguinis* strains from experimentally infected rabbits.

	Vegetation^a^		Blood^a^
Strain	Total CFU	CFU/g	CFU/ml
JFP36	1.7×10^9^	8.1×10^9^	1.1×10^4^
	(5.1×10^8^–3.3×10^9^)	(2.1×10^9^–9.3×10^9^)	(3.2×10^3^–2.5×10^4^)
*comCDE*	1.9×10^9^	6.4×10^9^	8.7×10^3^
	(6.2×10^8^–3.5×10^9^)^b^	(3.3×10^9^–8.9×10^9^)^b^	(8.3×10^2^–1.6×10^4^)^b^
*comX*	2.8×10^8^	2.4×10^9^	5.1×10^2^
	(8.9×10^7^–2.4×10^9^)^b^	(1.2×10^9^–7.6×10^9^)^b^	(3.9×10^2^–8.2×10^3^)^b^

a Median, (interquartile). N = 7 rabbits, except as indicated.

b N = 6.

**Figure 2:**
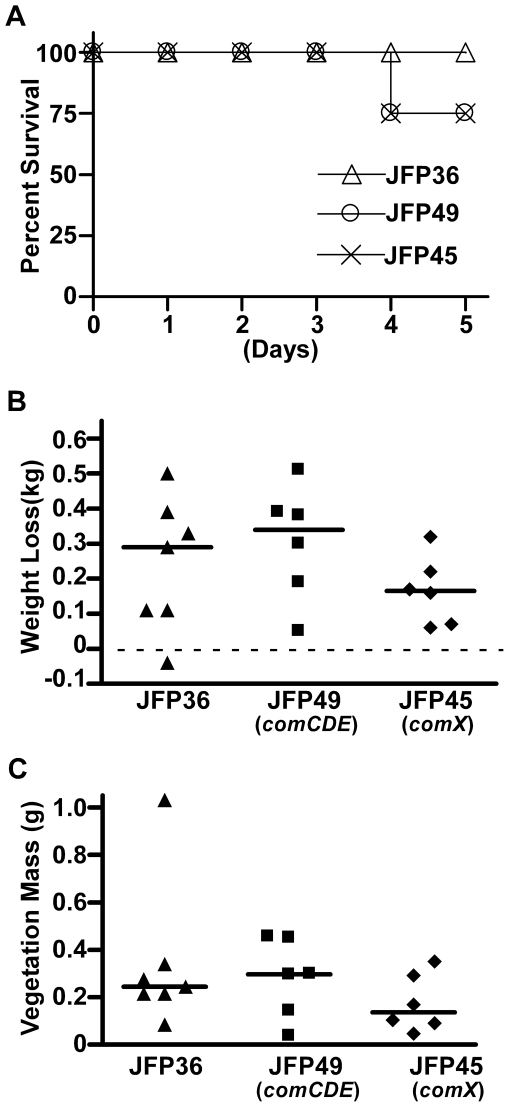
Assessment of IE pathology in rabbits inoculated with JFP36 or *com* mutants. Rabbits were inoculated with 10^8^ CFU of JFP36, JFP49 (*comCDE*), or JFP45 (*comX*). Data were combined from two separate experiments, each of which included all three strains. (A) Survival curve. (B) Total weight loss 5 days post-infection. Horizontal dashed line represents no weight loss. (C) Mass of aortic valve vegetations 5 days post-infection. For B and C, each symbol represents the value from a single rabbit, and horizontal lines indicated median values. No significant differences among strains were observed in any of the analyses.

We also examined pathology resulting from infection by comparing mortality, weight loss, and vegetation mass among the groups of rabbits. As mentioned above, two rabbits each inoculated with *comCDE* and *comX* mutants died on day four, while all rabbits infected with JFP36 survived until day 5 (Fig. 2A). Kaplan-Meier survival analysis indicated there was no significant difference in mortality among the groups. Since weight loss has previously been shown to be a reliable indicator of IE severity in a rabbit model [27], infected rabbits were weighed daily throughout the study. As shown in Fig. 2B, total weight loss after five days of infection did not differ significantly among rabbits infected with each strain. Likewise, no significant differences among groups were observed on days 1–4 post-infection (data not shown). Fig. 2C shows the masses of vegetations recovered at the end of the five-day infection. Our results indicated no significant differences in vegetation mass for rabbits infected with each of the three strains. Further, no consistent morphological differences were observed upon gross examination of the vegetations (data not shown). Taken together, our results suggest that the *S. sanguinis com* regulon is not required for IE virulence or pathology in the rabbit model under a variety of assay conditions.

## Discussion

As expected, deletion of either *comCDE*, which activates the early *com* genes, or *comX*, which is required for the late response, produced the identical phenotype of eliminating competence in *S. sanguinis*. We did not want to assume that the same would be true with regard to *com* regulon functions other than genetic competence. A number of early *com* regulon genes with functions involving cell lysis, virulence and biofilm formation have been identified in other streptococcal species, and some have been shown to be expressed in a *comX* mutant [16], [28], [33]. If one of these genes encodes a virulence factor, *comCDE* mutation would be expected to reduce virulence while a *comX* mutation would not. Conversely, instances of activation of ComX-dependent genes in the absence of *comCDE* have also been reported [34]–[37]. Thus, a ComX-dependent virulence gene might be expressed in a *comCDE* mutant, but not in a *comX* mutant. Finally, different *comD* mutations have been shown to either increase or decrease *S. pneumoniae* colonization depending on their effect on *comE* expression [38]. We therefore chose to examine virulence in both a mutant deleted for the entire *comCDE* operon and in a mutant deleted for *comX*.

Using a variety of assays and measures, we determined that neither *comCDE* nor *comX*-dependent genes were required for *S. sanguinis* IE virulence in the most commonly used model for this disease—the rabbit. This was in contrast to our previous findings in two studies showing reduced competitiveness of SK36 mutants in our standard rabbit model. The first of these used signature-tagged mutagenesis to identify 38 mutants that appeared to grow normally *in vitro*, yet possess reduced competitiveness *in vivo* [8]. Five of these were examined in CI assays, and four had CI values ranging from 0.31 to 0.0063 that were significantly less than 1.0. The second study identified additional mutants with significantly reduced CI values, including one with a mean CI of 2.9×10^−4^ [10]. Thus, the standard model identical to that used here was clearly capable of identifying mutants with reduced competitiveness. Finally, we have not tested these mutants in any other model, and it is certainly the case that different results might be obtained if we did. We note, however, that our earlier signature-tagged mutagenesis study also compared 40 *S. sanguinis* mutants in the rabbit and rat models, and found that the two models produced similar results except that the rat model was far less reproducible [8].

Our study appears to be the first to examine the effect of *comX* mutation on streptococcal virulence for any disease, although ComX-dependent genes encoding competence protein CoiA, a choline-binding protein CbpD, and the LytA autolysin were identified as contributing to virulence for pneumonia in a mutant screen of *S. pneumoniae* [19]. Comparison of the virulence of a *comX* mutant to strains possessing mutations in each of these genes and in *comCDE* could clarify the role of the *comX* gene for virulence in *S. pneumoniae*.

As far as we are aware, the effect of *comC*, *comD*, *or comE* deletion on virulence has been examined previously in only two other streptococcal species, *S. mutans* and *S. pneumoniae*. A *comCDE* mutant of *S. mutans* was shown to possess reduced cariogenicity in a rat model, but the reason for this effect was not determined [20]. Given the distinct nature of caries formation, which requires adherence to teeth, acid production, and acid tolerance, studies of *S. pneumoniae com* mutants likely provide a more relevant comparison to *S. sanguinis* and IE. In *S. pneumoniae*, studies employing *comD* mutants have reported attenuated virulence in murine models of respiratory tract infection [17]–[19], systemic infection [18], and bacteremia [17]. As with *S. mutans*, the mechanisms by which *comD* contributes to virulence in *S. pneumoniae* are not known. It has been shown that *com* regulon induction in *S. pneumoniae* induces cell lysis [39], [40]. It has also been shown that *comCDE*-dependent cell lysis in *S. pneumoniae* results in release of the cytoplasmic virulence factor pneumolysin [39]. This is likely mediated at least in part by the major autolysin, LytA, encoded by a *com* regulon gene [16], [41]. This could explain not only the reduced virulence of these mutants in assays examining pathology or death, but also the increased fitness for asymptomatic upper respiratory tract carriage of a *comE* mutant [38], since in this environment, it may be beneficial to *S. pneumoniae* to avoid damaging host tissue. It is not clear whether *com*-dependent lysis occurs in *S. sanguinis* or not, but it was interesting to note the slight (though insignificant) advantage of the *S. sanguinis comCDE* mutant in the competition assay. This was accompanied by a slight increase in growth of this mutant in BHI broth culture compared to SK36 or JFP36 (data not shown). It is possible that this was the result of *comCDE*-dependent cell lysis in the wild-type strains. This could suggest that IE virulence in *S. sanguinis* is more akin to *S. pneumoniae* colonization than to pneumonia or other invasive diseases. This would be consistent with our findings and with previous studies indicating that streptococcal endocarditis appears to mainly involve progressive enlargement of the infected vegetation by accrual of bacteria, platelets, and fibrin [7], [27], as opposed to the myocardial abscess formation that often occurs with other IE pathogens such as *Staphylococcus aureus* [4]. This would also be consistent with a recent finding that an endocarditis isolate of *S. sanguinis* was defective for competence [42]. In summary, then, *com* regulon activities apparently required for virulence in *S. mutans* and *S. pneumoniae* may also occur in *S. sanguinis* but appear to play no role in IE virulence in our model. It would be of interest to examine a *comCDE* mutant of *Streptococcus mitis* for IE virulence since this species is similar to *S. sanguinis* with regard to frequent isolation from IE cases, but is phylogenetically closer to *S. pneumoniae* and possesses a pneumolysin ortholog [43].

Our study was also useful for defining the limits of the rabbit IE model. We found that inoculum levels of 10^5^ to 10^6^ CFU were insufficient to ensure infection. Although this would not preclude their use in ID_50_ studies, larger inocula are clearly required to obtain meaningful results from competition assays. We also explored the use of infection periods longer than the 2 and 20-hr periods we have employed previously in this model [8]–[11]. Four deaths occurred prior to the end of study, all on day four. This is in contrast to the 20-hr infections performed here, in which no animals died prematurely, as well as our previous experiments with this model, in which premature deaths have been exceedingly rare (unpublished data). Upon necropsy, we observed that vegetations were far larger than we typically observe, with a median value of 276 mg in this study compared to 51 mg for a previous study [11] employing a 20-hr infection (*P* = 0.0006; Mann-Whitney test). This may have caused congestive heart failure by occluding blood flow, or have resulted in embolic stroke. We therefore suggest that any future studies with this model be limited to three days' duration, at least when applied to *S. sanguinis* SK36.
